# A Novel Phage Infecting the Marine Photoheterotrophic Bacterium *Citromicrobium bathyomarinum*

**DOI:** 10.3390/v14030512

**Published:** 2022-03-02

**Authors:** Ruijie Ma, Shuai Shao, Shuzhen Wei, Junlei Ye, Yahui Yang, Nianzhi Jiao, Rui Zhang

**Affiliations:** 1 State Key Laboratory of Marine Environmental Science, Fujian Key Laboratory of Marine Carbon Sequestration, College of Ocean and Earth Sciences, Xiamen University, Xiamen 361102, China; maruijie@stu.xmu.edu.cn (R.M.); weisz@stu.xmu.edu.cn (S.W.); yahuiyang@stu.xmu.edu.cn (Y.Y.); jiao@xmu.edu.cn (N.J.); 2 College of Ocean and Earth Sciences, Xiamen University, Xiamen 361102, China; shaoshuai_academic@163.com (S.S.); yejunlei@stu.xmu.edu.cn (J.Y.); 3 Southern Marine Science and Engineering Guangdong Laboratory, Zhuhai 519080, China

**Keywords:** bacteriophage, *Citromicrobium*, photoheterotrophic bacteria, auxiliary metabolic genes, viral tRNA

## Abstract

This study isolated and characterized a new phage infecting the marine photoheterotrophic bacterium *Citromicrobium bathyomarinum*, which fills the gap in research on phages targeting this ecologically important species. The phage vB_CbaS-RXM (RXM) has a dsDNA genome with a length of 104,206 bp and G+C content of 61.64%. The taxonomic analysis found a close evolutionary relationship between RXM, *Erythrobacter* phage vB_EliS-L02, and *Sphingobium* phage Lacusarx, and we propose that RXM represents a new species of the *Lacusarxvirus* genus. A one-step growth curve revealed a burst size of 75 plaque-forming units (PFUs) per cell in a 3-hour infection cycle. The lysis profile of RXM showed an intraspecific lethal rate of 26.3% against 38 citromicrobial strains. RXM contains 15 auxiliary metabolic genes (AMGs) related to diverse cellular processes, such as putative metabolic innovation and hijacking of host nucleotide metabolism to enhance its biosynthetic capacity. An in-depth analysis showed that phage functional genes strongly rely on the host for translation, while the translation of unique phage genes with less host dependency may be complemented by phage tRNA. Overall, our study investigated the infection kinetics, genetic traits, taxonomy, and predicted roles of AMGs and tRNA genes of this new phage, which contributes to a better understanding of phage diversity and phage–bacterium interactions.

## 1. Introduction

As the most abundant organisms in the ocean, marine viruses have attracted extensive attention. Virus-mediated lysis is considered important in the mortality of organisms and cycles of biogenic elements in marine ecosystems [1]. Due to frequent recombination, viruses undergo rapid gain and loss of genes and represent the largest reservoir of genetic diversity yet remain largely unexplored. Despite progress in high-throughput techniques, the isolation and characterization of new viruses are still an important method to continually decode viral dark matter from metagenomic data and understand novel information on specific virus–host interactions.

*Citromicrobium bathyomarinum* is a Gram-negative marine species in the family *Sphingomonadaceae* and the class *Alphaproteobacteria*. As it benefits from its good adaptability to dynamic environmental conditions [2], the species has been commonly found from various depths in seawater [3,4]. *C. bathyomarinum* is also an aerobic anoxygenic photoheterotroph that plays an important role in oceanic carbon cycling and energy flow [5]. Only by integrating information on phages targeting this species can we better understand how bacteriophages influence the ecology of citromicrobial photoheterotrophs. However, our current understanding of citromicrobial phages remains in its infancy. To date, only one ssDNA viral isolate infecting *C. bathyomarinum* RCC1878 has been reported [6], which is far behind the progress of other marine phages, such as phages infecting *Vibrio*, *Roseobacter*, and *Cyanobacteria*.

In this study, we reported a newly isolated phage, vB_CbaS-RXM, using *C. bathyomarinum* strain JL1366 as a trapping host. We conducted experiments to understand its infection kinetics, performed proteomic and genomic analyses to interpret phage genetic traits and taxonomy, and performed an in-depth analysis of phage AMGs and tRNA genes to explore phage–bacterium interactions.

## 2. Materials and Methods

### 2.1. Phage Isolation and Purification

*C. bathyomarinum* JL1366, an isolate from a seawater sample collected at a depth of 50 m in the South China Sea (17.99°N, 120.29°E), was used as a trapping host in this study. It was grown in a rich organic (RO) medium [2] with a shaking speed of 160 rpm/min at 28 °C. Using the double-layer agar method, we attempted to isolate infectious phages from various virus-containing samples collected from coastal and open-ocean seawater as well as sewage from different aquatic markets and farms. After five purification steps, the well-separated plaque was removed and stored in the storage medium (SM) (50 mM Tris-HCl, 0.1 M NaCl, and 8 mM MgSO_4_ (pH 7.59)) for subsequent experiments.

We amplified virions in 1 L of early log-phase host culture to prepare a high-titer phage solution. Lysates were centrifuged (10,000× *g*, 30 min, 4 °C) and passed through 0.22 μm membranes (Millipore, MA, USA) to remove cellular debris and polyethylene glycol 8000 (10% *w/v*) was added to precipitate virions. Viral particles were subsequently collected by centrifugation (10,000× *g*, 60 min, 4 °C), resuspended in 6 mL of SM, and further purified using cesium chloride gradient ultracentrifugation (1.3, 1.5, and 1.7 g/mL layers) with an Optima L-100 XP Ultracentrifuge (Beckman Coulter, California, United States) at 200,000× *g* for 24 h at 4 °C. The visible phage band was extracted and dialyzed through 30 kDa super-filters (Millipore, MA, USA) to remove CsCl.

### 2.2. Transmission Electron Microscopy (TEM)

Twenty microliters of the desalted phage solution were spotted on carbon-coated copper grids (200 mesh). After 30 min of adsorption in the dark, the phage sample was negatively stained with 1% phosphotungstic acid for 1 min, followed by air drying for 10 min. Phage images were captured using a JEM-2100 transmission electron microscope (JEOL, Tokyo, Japan) at 80 kV. The size of phage particles was measured from at least five TEM images using ImageJ software [7].

### 2.3. Determination of the Host Range

A collection of 38 citromicrobial strains isolated from various marine habitats and preserved in our laboratory was used in our host range test. The spot assay was applied as described in detail elsewhere [8]. Briefly, 5 μL of 1:100 phage serial dilutions were spotted on the bacterial lawn of each of 38 citromicrobial strains. Agar plates were sealed and incubated at 28 °C. The presence of phage plaques was inspected after 48 hours.

### 2.4. The One-Step Growth Curve

One milliliter of early log-phase host culture was exposed to virions at a multiplicity of infection of approximately 0.001, after which the mixture was immediately placed in the dark for 5 min to promote viral adsorption, and those virions that had not been adsorbed were removed by centrifugation. Cell pellets were washed and resuspended in 100 mL of fresh RO medium, and the culture was then incubated at 28 °C with a shaking speed of 160 rpm/min. Every 15 or 30 min, viral abundance was measured using the double-layer agar method. Viral burst size was calculated as the ratio between the number of virions at the growth plateau and the initial number of infected host cells [8].

### 2.5. Phage DNA Extraction, Sequencing and In Silico Analyses

Phage DNA was extracted using a phenol–chloroform–isoamyl alcohol method as previously described in detail [8] and sequenced using the Illumina Nova platform with a 300-bp paired-end DNA library. The filtered reads were then assembled de novo using Newbler assembler 2.8 [9] to generate the final assembled sequence. Genomic coverage and terminal redundancy were analyzed using PhageTerm version 1.0.12 [10].

Phage predicted genes were determined by combining the results of Prodigal (version 2.6.3) [11], MetaGeneAnnotator (version 1.0-0) [12], and the online GeneMarkS server [13]. Predicted genes were further annotated with BLASTp, Virfam [14], NCBI conserved domain search [15], and HHpred [16], with a cutoff of E-value < 10^−3^. The genome map was drawn with a custom Javascript. Genes for tRNAs were detected using tRNAscan-SE [17]. Genome-wide tBLASTx comparisons were conducted and visualized using Circoletto [18].

### 2.6. Structural Proteome and Data Processing

In order to identify the phage structural proteins from purified phage particles, fifty microliters of CsCl-purified phage solution was mixed with 100 μL of SDT lysis buffer (4% *w/v* SDS, 0.1 M DTT, and 100 mM Tris-HCl (pH 7.6)), and the mixture was incubated in a boiling water bath for 10 min and then separated on a standard SDS–PAGE gel. The SDS–PAGE gel slice was then excised, trypsinized, and analyzed using liquid chromatography–electrospray ionization–tandem mass spectrometry (LC-ESI-MS/MS). The experiment was performed on a Q Exactive Plus mass spectrometer (Thermo Fisher Scientific, Waltham, MA, USA) coupled to Easy nLC (Proxeon Biosystems, Odense, Denmark). Peptides were separated on a C18 reversed-phase column (100 × 0.075 mm × 3 μm; Thermo Fisher Scientific, Waltham, MA, USA) over 70 min at a flow rate of 250 nL/min with a linear gradient of increasing acetonitrile from 1% to 27%. The mass spectrometer was operated in data-dependent mode; a high resolution (70,000) MS scan (300–1800 m/z) was performed to select the most abundant precursor ions.

The MS data were analyzed and searched against a complete amino acid profile of RXM using MaxQuant version 1.6.4.0 [19]. The search followed an enzymatic cleavage rule of Trypsin/P and allowed maximal two missed cleavage sites and a mass tolerance of 20 ppm for fragment ions. Carbamidomethylation of cysteines was defined as fixed modification, while protein N-terminal acetylation and methionine oxidation were defined as variable modifications for database searching. The cutoff of the global false discovery rate for peptide and protein identification was set to 0.01.

### 2.7. Phylogenetic Analysis

Two phylogenetic trees were constructed to infer the taxonomy of phage RXM. First, the amino acid sequence of a canonical phage hallmark, terminase large subunits (TerL), was searched against the NCBI nonredundant database to retrieve protein homologs. The sequences were then submitted to Mega X [20] to build a neighbor-joining phylogenic tree with 1000 bootstraps. Second, complete amino acid profiles of RXM and other related phages were submitted to the VICTOR server (https://ggdc.dsmz.de/victor.php; accessed on 10 November 2021) for whole-genome tree building, which employs the genome BLAST distance phylogeny (GBDP) method [21] using the settings recommended for prokaryotic viruses [22]. The phylogenic tree was visualized and manipulated using iTOL v6 [23].

### 2.8. Calculation of Codon Usage (CU) and tRNA Relative Contribution Index (tRCI)

The CU profile of each phage gene and the host genome was determined using the Countcodon program (http://www.kazusa.or.jp/codon/cgi-bin/countcodon.cgi; accessed on 10 November 2021). The cosine similarity distance between the CU vector of each viral gene and the host genome was calculated [24], sorted from high to low, and categorized into 6 ordered bins (26 genes per bin). The value interprets the CU adaptation of each RXM gene to its host genome, with a value closer to 1 indicating better adaptation. The tRCI was calculated to assess the potential contribution of this tRNA to the translation efficiency of each phage gene [24]. Likewise, tRCI values were sorted and categorized, and genes with a larger tRCI value were predicted to benefit more from phage tRNA. For each bin, genes from each functional category were counted, and gene enrichment and underrepresentation were considered significant if *p* < 0.05, which was determined using the hypergeometric distribution probability.

## 3. Results and Discussion

### 3.1. Biological Features of RXM

In our attempts to isolate novel phages targeting shallow sea-dwelling *C. bathyomarinum* strain JL1366, we screened seawater samples from coastal (Jiulong and Changjiang Estuaries) and open-ocean environments (South China Sea and western Pacific), as well as water samples from different aquatic markets and farms (cities Xiamen, Zhangzhou, and Guangzhou), against exponentially growing JL1366 cultures. We isolated a new phage, RXM, from sewage collected at a seafood market in Zhangzhou city, China (23.70°N, 117.43°E).

**Figure 1:**
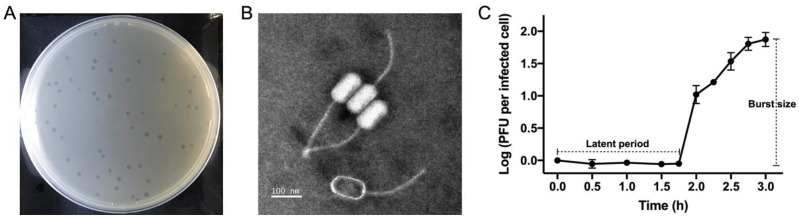
(**A**) Plaques of RXM formed on the bacterial lawn of *C. bathyomarinum* JL1366 after 24 hours of incubation. As a reference, the diameter of the culture dish was 90 mm. (**B**) TEM image of RXM. Scale bar, 100 nm. The phage characterized by the empty capsid structure indicates a lack of DNA, which is probably due to the damage during sample preparation. (**C**) One-step growth curve of phage RXM and each data point is shown as the mean ± SD of three independent replicates.

Plaques of phage RXM are clear and round, with a well-defined boundary ranging from 1.5 to 1.8 mm in diameter (Figure 1A). TEM inspection revealed that RXM displays a unique *Siphoviridae* B3 morphotype [25], with a prolate capsid (111.50 ± 1.48 nm long and 56.02 ± 3.06 nm wide) and a long flexible tail (220.57 ± 3.88 nm long). Intriguingly, TEM showed that virions often adhere to each other through their heads (Figure 1B; see also Supplementary Figure S1A and S1B). We noted that similar aggregates are observed in *Rhizobiales* phage phiJL001 [26], which has a B2 morphotype (B2, *Siphoviridae* with a prolate capsid; B3, *Siphoviridae* with a remarkably prolate capsid). We speculate that these phage aggregates may increase the likelihood of adsorption and attachment during the initial stage of infection.

**Figure 2:**
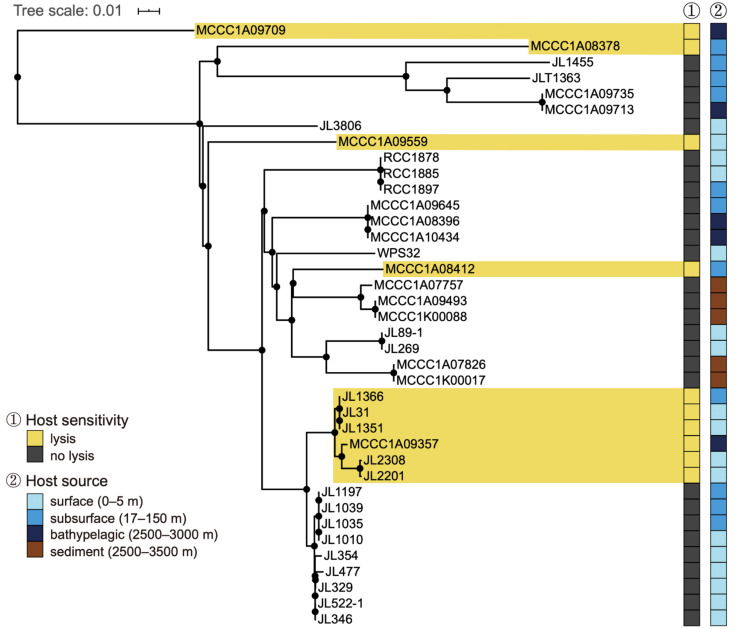
Intraspecific lysis profiles of phage RXM against 38 citromicrobial strains recovered from various seawater depths. Citromicrobial whole-genome phylogenic tree was built using progressive Mauve [28]. Sensitive strains are highlighted using yellow squares, and insensitive strains are highlighted with dark brown squares.

The one-step growth curve revealed a latent period of 105 min, a rise period of 75 min, and burst size (the fold increase in phage concentration) of approximately 75 PFUs per cell (Figure 1C). Overall, approximately 3 hours were required for RXM to complete a round of propagation in the host cell. The host range test included a total of 38 citromicrobial strains from various marine environments (Supplementary Table S1). These strains share a pairwise 16S rRNA gene sequence identity >98%, and hence our large survey well reflects an intraspecific lysis profile of phage RXM. The results showed that RXM lyses 10 of 38 tested strains, with an intraspecific lethal rate of 26.3%. Sensitive hosts include isolates from the surface, subsurface, and deep-sea samples, indicating a corresponding wide distribution of RXM in the ocean. Notably, phage RXM lysed the lineage containing JL1366 (the trapping host) and five relative strains while failing to kill the sibling lineage (Figure 2), members of which display a whole-genome average nucleotide identity of >99% compared to JL1366. Such different sensitivities towards a single phage between highly similar intraspecific individuals are likely due to phage–bacterium coevolutionary arms races. For example, the phage may expand its host spectrum through the alteration of host-specific tail-related genes. Alternatively, the host bacterium can alter its surface proteins to block phage attachments [27].

### 3.2. Genomic Features of RXM

**Figure 3:**
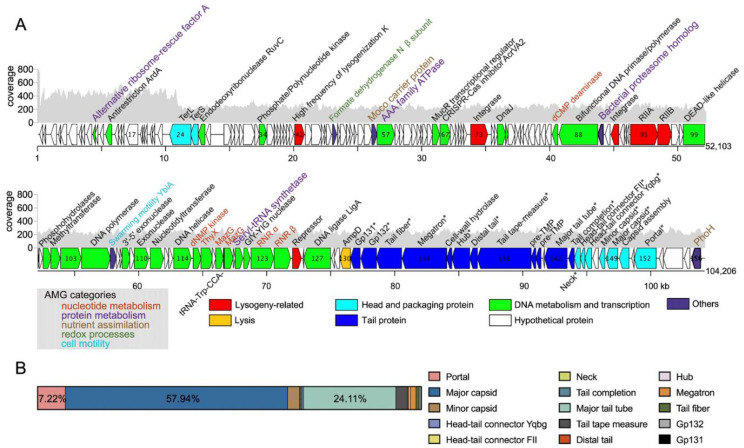
Genomic and proteomic features of phage RXM. (**A**) Full-genome map of RXM. Mean fold coverage at each position is shown using gray bars. The orientation of each predicted gene product corresponds to the direction of transcription. Genes within different functional categories are indicated by the colors noted below. Predicted phage AMGs are marked with different colors based on different functional categories displayed in the lower-left quadrant. Confirmed viral structural genes are marked with an asterisk (*). (**B**) Relative abundance of RXM structural proteins detected using LC-ESI-MS/MS.

Sequencing produced a circular assembly with a length of 104,206 bp. The large genome is consistent with its B3 morphotype [25], and the extremely prolate capsid of an enlarged size is able to carry a larger genome than its icosahedral counterpart. The first 10,992 bp were determined to be a direct terminal repeat (DTR), with a 434.36 ± 68.26× sequencing coverage that was approximately 2-fold greater than that of the rest of the genome (230.88 ± 28.14×) (Figure 3A). The fold change values indicate that two copies of DTR are present in one completely packaged genome. Therefore, we determined the physical end of the RXM as the boundary of the DTR.

RXM has a dsDNA genome with a G + C content of 61.64%, similar to that of the trapping host (65.07%). A total of 156 genes were identified and annotated (Supplementary Dataset S1). A tRNA-Trp gene with anticodon CCA was predicted from the RXM genome (Supplementary Figure S2). Structural proteomics analysis was conducted to examine genes constructing the RXM virion and confirm our genome-based gene predictions (Figure 3B and Figure S3; see also Supplementary Table S2). Fifteen structural components were identified, among which two were previously annotated as hypothetical (Gp131 and Gp132). As the two genes are located adjacent to the tail-related module, we regarded them as novel tail-related genes.

For most phage types, the lysis genes, such as genes for the holin, endolysin, and spanin subunits, often cluster in a lysis cassette [29]. However, phage RXM only encodes an *N*-acetylmuramoyl-L-alanine amidase (AmpD), which is also known as a peptidoglycan amidohydrolase involved in host lysis. The *ampD* gene is adjacent to two upstream structural tail genes and two downstream genes encoding hypothetical proteins. No significantly similar sequences for these two hypothetical proteins were identified from BLASTp, NCBI conserved domain database, and HHpred. Thus, such a lysis gene cassette may represent a novel system. Meanwhile, six scattered lysogeny-related genes were identified, including two canonical hallmarks (integrase and repressor) of lysogeny (Figure 3A). Thus, RXM or its ancestor may possess lysogeny potential, in which the phage establishes a symbiotic relationship with the host as an integrated (lysogenic) or nonintegrated prophage (pseudolysogenic).

### 3.3. RXM Represents a New Species of the Lacusarxvirus Genus

**Figure 4:**
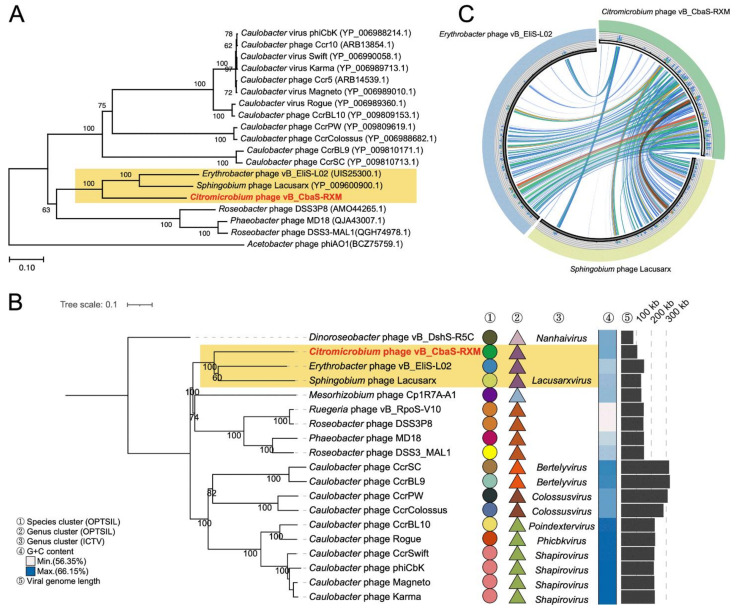
Evidence supporting the taxonomy of RXM. (**A**) The neighbor-joining phylogenic tree based on amino acid sequences of phage TerL from RXM and related phages. Bootstrap values were based on 1000 replicates. (**B**) GBDP tree of RXM and related phages. The taxonomic assignment from OPTSIL is attached for reference. Information on viral genomic traits is also presented. ICTV, the International Committee on Taxonomy of Viruses. (**C**) Genome-wide comparison of RXM with *Sphingobium* phage Lacusarx and *Erythrobacter* phage vB_EliS-L02. Each segment represents an individual genome, and the ribbons connecting genomes represent local alignments produced by tBLASTx. Blue, green, orange, and red ribbons represent 25% blocks up to the maximum bitscore of 100%, respectively.

We conducted a marker-gene survey using phage TerL and a GBDP tree based on complete phage amino acid profiles to understand the taxonomy of phage RXM (Figure 4A,B). Both approaches consistently show that RXM forms a tightly cohesive group with *Erythrobacter* phage vB_EliS-L02 (GenBank OL955261.1) and *Sphingobium* phage Lacusarx [30] (GenBank KY629563.1) of the *Lacusarxvirus* genus, with reliable bootstrap values. Host strains of the three phages all belong to the *Sphingomonadales* order of the class *Alphaproteobacteria*. The OPTSIL taxonomic assignment (http://www.goeker.org/mg/clustering/; accessed on 22 February 2022) suggested that the three phages are grouped into a single genus but should be regarded as different species (Figure 4B). We also investigated the genome-wide tBLASTx similarity between the three phages and observed good synteny (Figure 4C). Indeed, the three members share certain features; for example, they all display similar B3 morphotypes, contain tRNA and lysogeny-related genes, and adopt the same packaging mode that results in two copies of DTR [30]. Thus, we propose here that phage RXM represents a new species of the *Lacusarxvirus* genus.

### 3.4. Phage–Host Interactions Inferred from Viral AMGs

As a phage containing a dsDNA genome exceeding 100 kb, RXM encodes an abundant number of AMGs that are predicted to regulate various cellular processes. Detailed annotations of all identified AMGs are provided in Supplementary Dataset S2.

#### 3.4.1. Nucleotide Metabolism

**Figure 5:**
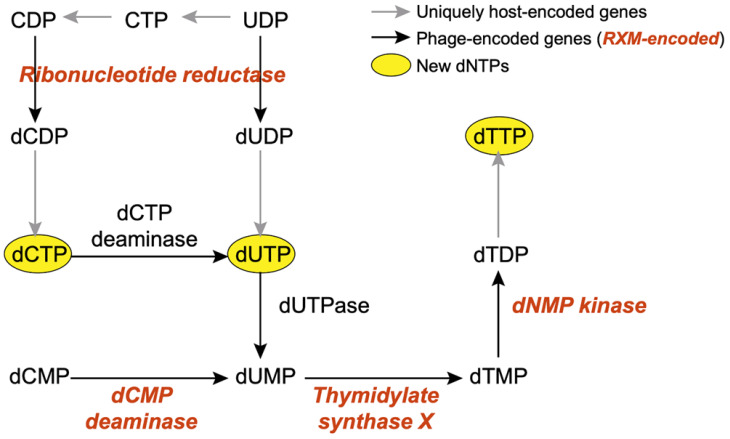
A diagram showing how phages hijack host nucleotide biosynthesis to enhance the viral biosynthetic capacity. Phages encode abundant genes (black arrows) to promote the production of new dNTPs (yellow ovals). In the RXM genome, a few related genes are recognized (highlighted in bold red italics).

Approximately half of RXM AMGs (7/15) are sorted into this category, and this finding is expected given the huge demand for host nucleotides for viruses with a large genome. RXM *g123* and *g124* encode the α and β subunits of ribonucleotide reductase (RNR), respectively, which converts ribonucleotides to deoxyribonucleotides during the transition from RNA to DNA [31]. Together with a chain of enzymes (Gp87, dCMP deaminase; Gp115, dNMP kinases; Gp116, thymidylate synthase X), RXM is able to hijack host nucleotide biosynthesis to enhance the viral biosynthetic capacity of new dNTPs (Figure 5). Moreover, RXM may scavenge host overrepresented C or G bases relative to its own bases through the MazG-encoding genes *g118* and *g119* [32].

#### 3.4.2. Protein Metabolism

RXM *g10* encodes alternative ribosome-rescue factor A (ArfA) that rescues undesired ribosome stalling due to aberrant mRNAs or transcriptional errors [33]. Meanwhile, RXM *g121* encodes seryl-tRNA synthetase (SerRS) that catalyzes the ligation of serine to its cognate tRNA-Ser for protein synthesis [34]. The *g89* encodes a bacterial proteasome homolog (BPH) that mediates protein degradation and enables rapid cellular responses to different environmental conditions. The refolding and degradation of proteins are powered by ATP hydrolysis conducted by ATPases associated with diverse cellular activities (RXM Gp57) [35].

#### 3.4.3. Nutrient Assimilation

The phage contains a phosphate (P) starvation-inducible protein PhoH that possibly regulates host P uptake and metabolism under low-P conditions (most open-ocean environments). PhoH also enhances phage fitness as it supplements P to meet demand in the late stage of viral infection. In addition, a molybdenum cofactor (Moco) carrier protein was identified. It is involved in the storage and transport of Moco, a major component in Mo-dependent enzymes that catalyze key metabolic reactions in global sulfur, nitrogen, and carbon cycles [36]. In addition, RXM Gp48 is similar to the β subunit of *Escherichia coli* formate dehydrogenase N (Fdn-N). Fdn-N is a key component of nitrate respiration, and its β subunit functions as a transmembrane anchor that enables electron transport [37]. Gp48 likely enables the phage to modulate the activity of host photosynthetic electron transport, which may contribute to host fitness, as its targeted citromicrobial hosts belong to photoheterotrophs.

#### 3.4.4. Swarming Motility

A homolog of YbiA-like swarming motility protein (Gp105) was identified. Previously, *ybiA* in temperate phage DLP4 was shown to restore the swarming motility activity of the *ybiA-*defective strain through lysogenic conversion [38]. Phage-encoded YbiA enhances host motility in surface-related environments and likely promotes a cellular response to adverse environmental factors. A similar scenario might also occur in RXM, which also contains several lysogeny-related genes.

### 3.5. Viral tRNA Facilitates the Translation of Unique Genes That Differ in CU from the Host tRNA Inventory

**Figure 6:**
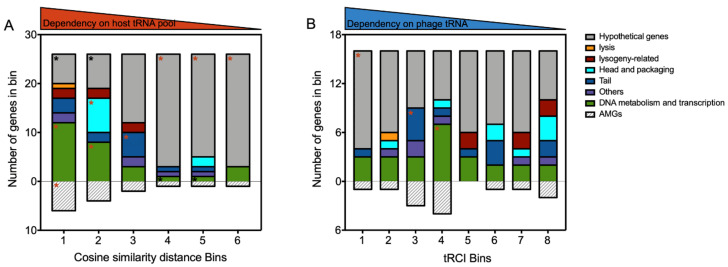
Phage gene contents in bins sorted by cosine similarity distance to host genome (**A**) and by tRCI values (**B**). Significant enrichment and underrepresentation (*p* < 0.05) of genes associated with specific functions are depicted with red and black asterisks (*), respectively.

The tRNA genes are known to be critical components in gene translation. Because RXM carries a single tRNA-Trp-CCA gene, we performed *in silico* analyses to infer the interplay between viral tRNA and the host tRNA pool. First, 156 RXM genes were sorted by their cosine similarity distance to the host genome in descending order and then arranged into six bins in an orderly manner (Figure 6A; see also Supplementary Dataset S3). Functional genes were significantly enriched among the top two bins (*p* = 0 and 0.0002) but underrepresented among the bottom three bins (*p* = 0.0007, 0.0108, and 0.0007). This tendency indicates that the expression of functional viral genes mainly relies on the host tRNA pool. Specifically, genes enriched in bins 1 and 2 are involved in DNA metabolism and transcription (*p* = 0.0002 and 0.0411), and bin 1 is enriched in viral AMGs (*p* = 0.0168). The observed CU adaptation priority of genes critical for replication and phage–host interactions reflects how phages maximize host resources during infection.

The tRCI of 128 viral genes containing the complementary codon UGG of tRNA-Trp-CCA was also calculated, sorted, and categorized to further estimate the contribution of phage tRNA to the translation of self genes (Figure 6B; see also Supplementary Dataset S3). Bin 1 is significantly enriched in 12 hypothetical viral proteins (*p* = 0.0472), 8 of which are also present in the bottom three cosine similarity distance bins. Based on this finding, RXM tRNA-Trp-CCA plays a significant role in complementing the expression of unique genes that differ in CU from the host tRNA inventory. Therefore, the dependence on host translation machinery may be reduced to some degree when the phage carries its own tRNA gene.

Taken together, viral functional genes likely adapt their CU patterns to match desired hosts, whereas viral tRNA complements the translation of unique genes with less host dependency. This strategy might enhance phage flexibility in changing environments or during host jumps. Our data are consistent with two previous studies examining tRNA-carrying phages infecting marine *Cyanobacteria* [24,39], suggesting that these findings may have broader implications for tRNA-carrying viruses.

## 4. Conclusions

This study isolated and characterized a new phage infecting the marine photoheterotrophic bacterium *Citromicrobium bathyomarinum*. Our data provide valuable information on the physiology and ecology of phages targeting this ecologically important species, which have been understudied thus far. Our findings also raise several important questions for future investigations: (i) Can the phage enter lysogeny, and if so, how will this lifestyle transition affect the host fitness and population dynamics? (ii) What are the determinants of the putative phage–bacterium coevolutionary arms races observed from the lysis profile of phage RXM? (iii) What is the driving factor for the variation in the number of tRNAs carried by the virus? For example, RXM has only one tRNA gene, while its homologs at the single genus level, *Erythrobacter* phage vB_EliS-L02 and *Sphingobium* phage Lacusarx, carried 26 and 27 predicted tRNAs, respectively. iv) What are the sources of identified phage AMGs, and how do these genes coevolve with the phage or the host bacterium? Overall, this study provides a new model system of viruses targeting marine citromicrobial photoheterotrophs, and future studies tackling these questions may provide more far-reaching insights into virus–host interactions.

## Data Availability

The complete genome of phage RXM was deposited in the NCBI GenBank database with the accession number OL963731.1.

## Supplementary Materials

The following supporting information can be downloaded at: https://www.mdpi.com/article/10.3390/v14030512/s1, Figure S1: TEM images of RXM virions. (A) Scale bar, 200 nm. (B) Scale bar, 100 nm; Figure S2: Predicted tRNA-Trp-CCA structure in phage RXM, with an internal score of 42.7. The tRNA gene has a length of 74 bp and is located between 66,594 and 66,667 bp of the phage RXM genome; Figure S3: SDS–PAGE picture of phage structure proteins. M—protein molecular weight marker. Table S1: Citromicrobial strains with pairwise 16S rRNA gene sequence identity >98% in the host range test and their susceptibility to RXM; Table S2: Mass spectrometry data for phage RXM. A minimum of two unique peptides and 5% sequence coverage were used as threshold values; Dataset S1: Genomic annotation of RXM; Dataset S2: Annotation of AMGs identified from the RXM genome; Dataset S3: Cosine similarity distance and tRCI values of each RXM gene.
