# Supplementary material for: A Novel Phage Infecting the Marine Photoheterotrophic Bacterium Citromicrobium bathyomarinum
Source: Viruses. 2022 Mar 2;14(3):512. doi: 10.3390/v14030512 (PMC8953757; doi:10.3390/v14030512)
Supplement: Supplementary file 1 [file viruses-14-00512-s001.zip › Supplementary Tables&Figures.pdf]

Supplementary Materials

Table S1. Citromicrobial strains with pairwise 16S rRNA gene sequence identity >98% in the host-range test and their susceptibility to RXM.

**Strain**[a]	**Latitude (°)**	**Longitude (°)**	**Isolation source**	**Depth (m)**	**Layer**	**Susceptibility to RXM**
JL31	24.4584	118.247	South China Sea	0	surface	+
JL1351	17.9936	120.2867	South China Sea	−5	surface	+
JL2201	−26.018	−13.8568	South Atlantic Ocean	0	surface	+
JL1366	17.9936	120.2867	South China Sea	−50	subsurface	+
JL2308	18.3924	161.3252	Pacific Ocean	0	surface	+
MCCC1A08378	−3.1027	−102.5535	Pacific Ocean	−25	subsurface	+
MCCC1A08412	−3.1027	−102.5535	Pacific Ocean	−70	subsurface	+
MCCC1A09357	−14.0491	−14.3844	South Atlantic Ocean	−2,927	bathypelagic	+
MCCC1A09559	−6.9878	88.9906	Indian Ocean	0	surface	+
MCCC1A09709	−15.2833	−13.6	South Atlantic Ocean	−2,700	bathypelagic	+
JL354	21.6837	112.9182	South China Sea	0	surface	-
JL477	22.1667	115.1528	South China Sea	0	surface	-
WPS32	17	115	South China Sea	0	surface	-
JLT1363	17.9936	120.2867	South China Sea	−50	subsurface	-
JL89-1	31.5082	122.9288	East China Sea	0	surface	-
JL269	31.5	122.18	East China Sea	0	surface	-
JL329	18.463	113.4912	South China Sea	0	surface	-

JL346	20.7533	115.2422	South China Sea	0	surface	-
JL522-1	22.2006	113.8024	South China Sea	0	surface	-
JL1010	21	125	Philippine Sea	0	surface	-
JL1035	2	130	Philippine Sea	−50	subsurface	-
JL1039	2	130	Philippine Sea	−50	subsurface	-
JL1197	2	130	Philippine Sea	−50	subsurface	-
JL1455	18.285	109.1072	South China Sea	−17	subsurface	-
JL3806	37.2181	119.8468	Bohai Sea, China	0	surface	-
MCCC1A07757	−26.0211	−13.8521	South Atlantic Ocean	−2,545	bathypelagic[S]	-

Table S1| Continued

**Strain[a]**	**Latitude (°)**	**Longitude (°)**	**Isolation source**	**Depth (m)**	**Layer**	**Susceptibility to RXM**
MCCC1A07826	−26.0189	−13.8537	South Atlantic Ocean	−2,562	sediment	-
MCCC1A08396	−3.1027	−102.5535	Pacific Ocean	−2,742	bathypelagic	-
MCCC1A09493	−13.3552	−14.3112	South Atlantic Ocean	−3,142	sediment	-
MCCC1A09645	−15.2833	−13.6	South Atlantic Ocean	−50	subsurface	-
MCCC1A09713	−15.2667	−13.6	South Atlantic Ocean	−2,700	bathypelagic	-
MCCC1A09735	−15.2833	−13.6	South Atlantic Ocean	−150	subsurface	-
MCCC1A10434	−15.0333	−13.0556	South Atlantic Ocean	−2,891	bathypelagic	-
MCCC1K00017	−3.1041	−102.5544	Pacific Ocean	−3,394	sediment	-
MCCC1K00088	14.7506	−44.9784	North Atlantic Ocean	−2,990	sediment	-
RCC1878	34.133	18.45	Mediterranean Sea	−5	surface	-

RCC1885	34.133	18.45	Mediterranean Sea	−5	surface	-
RCC1897	38.633	7.917	Mediterranean Sea	-85	subsurface	-

[a]Strains with the prefix ‘JL/WPS’, ‘MCCC’, and ‘RCC’ were obtained from the Center for Collection of Marine Bacteria (CCMB), the Marine Culture Collection of China (MCCC) and the Roscoff Culture Collection (RCC), respectively;

+, sensitive; -, insensitive.

Table S2. Mass spectrometry data for phage RXM. A minimum of two unique peptides and 5% sequence coverage were used as threshold values.

ORF	Putative function	Mol. weight [kDa]	No. of peptides	No. of unique peptides	Sequence coverage (%)	Relative abundance (%)
131	Hypothetical protein	27.622	7	7	34	0.14
132	Hypothetical protein	44.618	9	9	24.5	0.44
133	Tail fiber	68.795	17	17	47.2	0.80
134	Megatron	125.61	32	32	32.8	1.52
136	Hub	51.356	11	11	33.7	0.39
137	Distal tail	22.608	8	8	43.1	0.40
138	Tail tape measure	151.21	59	59	44.9	2.95
142	Major tail tube	62.059	29	29	58.4	24.11
143	Tail completion	16.535	8	8	40	0.46
144	Neck	17.589	2	2	13	0.05
145	Head-tail connector FII	13.852	4	4	35.8	0.27
146	Head-tail connector Yqbg	18.631	4	4	27.8	0.20
148	Minor capsid	15.388	5	5	51.4	3.12
149	Major capsid	33.165	33	33	98.7	57.94
152	Portal	65.075	33	33	61.6	7.22

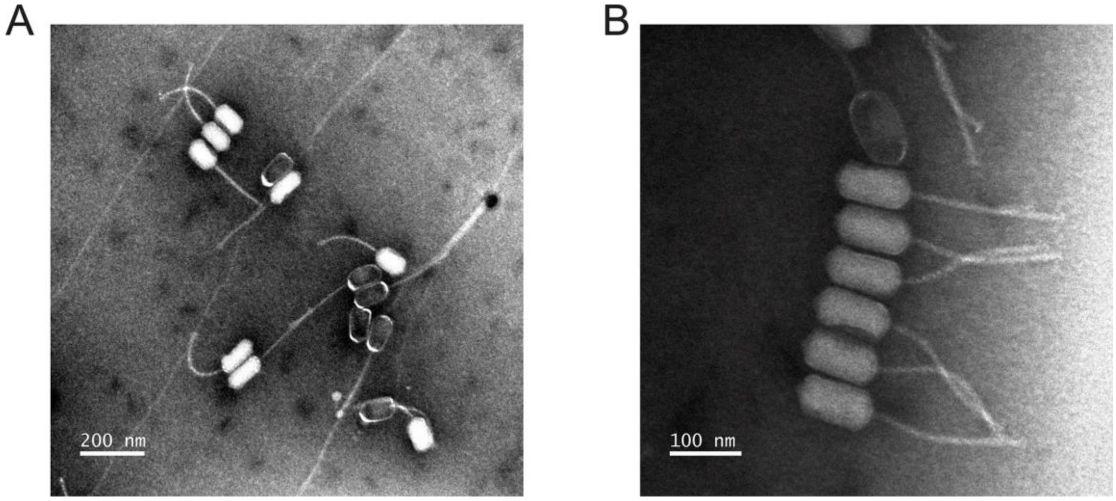

Figure S1. TEM images of RXM virions. (A) Scale bar, 200 nm. (B) Scale bar, 100 nm.

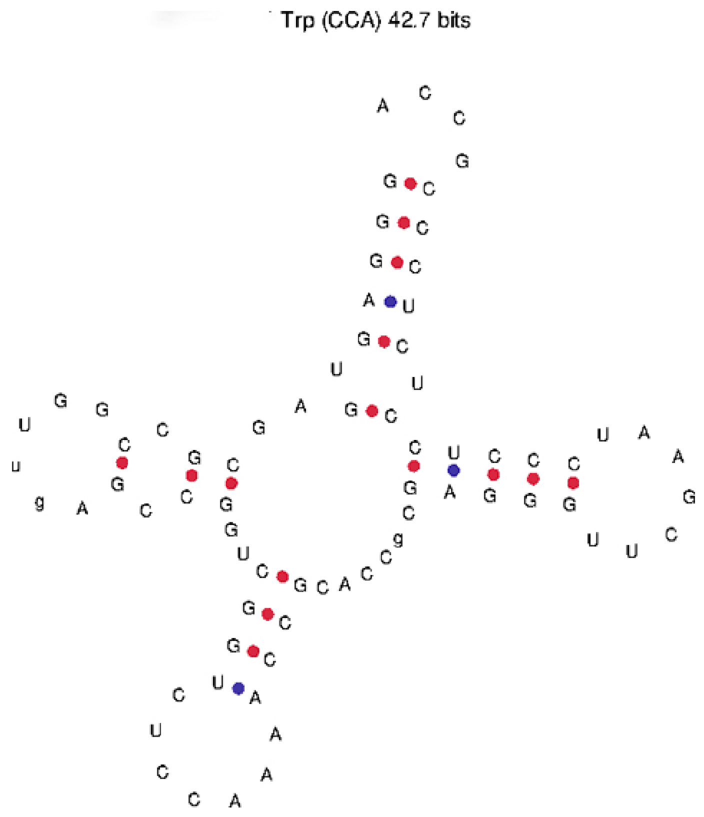

Figure S2. Predicted tRNA-Trp-CCA structure in phage RXM, with an internal score of 42.7. The tRNA gene has a length of 74 bp and is located between 66,594-66,667 bp of the phage RXM genome.

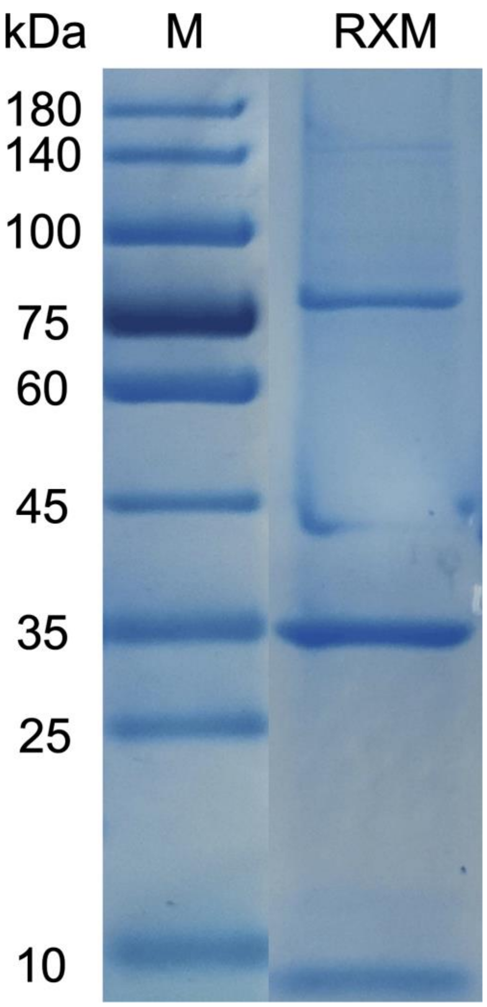

Figure S3. SDS–PAGE picture of phage structure proteins. M—protein molecular weight marker.
